# Occupational and environmental hazard assessments for the isolation, purification and toxicity testing of cyanobacterial toxins

**DOI:** 10.1186/1476-069X-8-52

**Published:** 2009-11-19

**Authors:** Ian Stewart, Wayne W Carmichael, Ross Sadler, Glenn B McGregor, Karen Reardon, Geoffrey K Eaglesham, Wasantha A Wickramasinghe, Alan A Seawright, Glen R Shaw

**Affiliations:** 1 Queensland Health Forensic and Scientific Services, 39 Kessels Road, Coopers Plains, Queensland 4108, Australia; 2 School of Public Health, Griffith University, Logan Campus, University Drive, Meadowbrook, Queensland 4131, Australia; 3 Department of Biological Sciences, Wright State University, 3640 Colonel Glen Highway, Dayton, Ohio 45435, USA; 4 Queensland Department of Environment and Resource Management, 120 Meiers Road, Indooroopilly, Queensland 4068, Australia; 5 The University of Queensland, National Research Centre for Environmental Toxicology, 39 Kessels Road, Coopers Plains, Queensland 4108, Australia; 6 School of Public Health, Griffith University, Gold Coast Campus, Parklands Drive, Southport, Queensland 4222, Australia; 7 Australian Rivers Institute, Griffith University, Gold Coast Campus, Parklands Drive, Southport, Queensland 4222, Australia

## Abstract

Cyanobacteria can produce groups of structurally and functionally unrelated but highly potent toxins. Cyanotoxins are used in multiple research endeavours, either for direct investigation of their toxicologic properties, or as functional analogues for various biochemical and physiological processes. This paper presents occupational safety guidelines and recommendations for personnel working in field, laboratory or industrial settings to produce and use purified cyanotoxins and toxic cyanobacteria, from bulk harvesting of bloom material, mass culture of laboratory isolates, through routine extraction, isolation and purification. Oral, inhalational, dermal and parenteral routes are all potential occupational exposure pathways during the various stages of cyanotoxin production and application. Investigation of toxicologic or pharmacologic properties using *in vivo *models may present specific risks if radiolabelled cyanotoxins are employed, and the potential for occupational exposure via the dermal route is heightened with the use of organic solvents as vehicles. Inter- and intra-national transport of living cyanobacteria for research purposes risks establishing feral microalgal populations, so disinfection of culture equipment and destruction of cells by autoclaving, incineration and/or chlorination is recommended in order to prevent viable cyanobacteria from escaping research or production facilities.

## Introduction

Over 40 species in some 20 cyanobacterial genera can produce a range of structurally and functionally diverse toxins, known as cyanotoxins. Freshwater and marine cyanobacteria, encompassing both planktonic and benthic forms, can produce potent toxins, some of which have been well characterised in terms of their toxic effects and some of which are less well-understood [1]. The known cyanotoxins can exert their toxic effects through a variety of mechanisms (depending on the particular toxin): some are potent neurotoxins that can cause respiratory dysfunction and death through paralysis of respiratory muscles, e.g. saxitoxins, anatoxin-a, anatoxin-a(S). Some affect the liver and other organs, e.g. microcystins, nodularin, cylindrospermopsin, and some exert acute irritant effects on the skin and mucous membranes, e.g. debromoaplysiatoxin, lyngbyatoxin A. Some cyanotoxins are potent tumour promoters, e.g. debromoaplysiatoxin, lyngbyatoxin A, microcystins, nodularin. Long-term, low-dose exposures to microcystins and/or cylindrospermopsin in drinking water are suspected (but so far unproven) risk factors for the development of some types of cancer. Cyanobacteria are rich sources of novel bioactive compounds, many of which are poorly researched with respect to toxicity [2].

**Figure 1:**
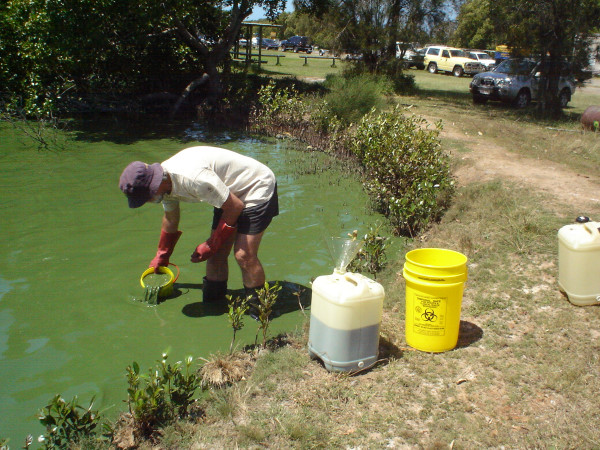
**Field collection of bloom material for cyanotoxin extraction**. Unobtrusive and common-sense personal protective equipment - waterproof boots and gloves in this case - may be used to harvest bloom biomass. Workers undertaking such activities should be familiar with the risks of accidental immersion, however, and care must be taken to ensure a secure footing. Photo by author WAW.

Several cyanotoxins have been chemically synthesised, e.g. anatoxin-a [3-5], cylindrospermopsins [6,7], saxitoxins [8,9], lyngbyatoxin A [10,11], aplysiatoxins [12], and one of the microcystins and some microcystin structural components [13,14]. However, the processes involved in synthesis of cyanotoxins like cylindrospermopsin and microcystins are complex and multiple steps are required, so those particular molecules are unlikely to be synthesised on a commercial scale in the near future. Therefore the most efficient methods for obtaining research-enabling quantities of many cyanotoxins in the short to medium turn will be to bulk-harvest cyanobacteria from natural waterbodies or to grow laboratory isolates in mass culture. Figure 1 shows field collection of bloom biomass for the purpose of extracting cyanotoxins (nodularin from *Nodularia spumigena *in this example). Although blooms such as in Figure 1 may attain very high densities, such conditions are not frequently encountered by staff engaged in field sampling for water quality monitoring purposes. Laboratory cultures grown for toxin production are likely to contain higher concentrations of specific toxins than are usually found in the field. Regular occupational handling of mass culture material would therefore be expected to present a greater human health risk should accidental exposures occur.

The hazard assessment guidelines presented in this manuscript are designed for use by scientific and technical staff involved in any of the various stages necessary to concentrate toxic cyanobacteria for the isolation of known cyanotoxins or natural products of unknown or uncertain toxicity, and subsequent extraction, isolation and purification of cyanotoxins for sale or for their own research programs. Exposure routes for actual and potential harmful effects are by inhalation, cutaneous contact, and oral or parenteral exposure. Toxic concentrations and doses will vary significantly depending on the particular cyanotoxin being processed and the exposure route, as will the expected health effects. In the absence of specific risk assessment guidelines for handling individual cyanotoxins, these recommendations should be regarded as preliminary and intentionally non-specific. This hazard assessment will present a precautionary approach for research and technical staff working to extract, isolate, concentrate and purify cyanobacterial toxins for research purposes. This document is not intended for use by workers exposed to cyanobacteria in other occupational settings such as sample collection of water or associated biota, or disposal of nuisance biomass; separate occupational health and safety guidelines are appropriate for such activities, e.g. see Metcalf et al [15] for environmental sampling protocols.

## Bulk harvesting of cyanobacteria: collection from natural waterbodies or laboratory mass culture

### Oral exposure risks

Harvesting of dense cyanobacterial biomass may result in higher short-term exposures than would be experienced during recreational activities. Most leisure-seekers avoid exposure to dense surface scums of planktonic cyanobacteria and floating mats of liberated benthic cyanobacteria, whereas such infestations will be the focus of attention for scientific staff. It follows that when collecting bloom biomass, the utmost care should be taken to avoid accidental immersion, as oral ingestion of contaminated water may occur by reflex swallowing. The associated risks are underlined by a well-documented case report concerning an individual who was planning to swim at a lake in Saskatchewan, Canada that was affected by a cyanobacteria bloom. He accidentally fell into the water, and swallowed an estimated half-pint (240 mL); within hours he developed an acute gastro-intestinal disorder which progressed to a flu-like illness characterised by fever, severe headache, arthralgia and myalgia [16]. It should be noted that sudden immersion stimulates reflex swallowing by the so-called immersion-deglutition reflex; the swallowing reflex may be more likely to occur following immersion in water with temperatures below 25°C [17,18]. Sudden accidental immersion in cold waters compared to warmer waters in temperate or tropical regions may present a relatively greater risk of reflex swallowing. However, occupational safety programs covering bulk harvesting of toxic or potentially toxic cyanobacteria should encompass measures to minimise the risk of accidental immersion at all latitudes.

Workers collecting cyanobacterial biomass by boat constitute a special case. Staff should therefore pay particular attention to prevention of accidental immersion and the subsequent potential for inadvertent oral exposure. Appropriate considerations are awareness of weather conditions, seaworthiness of the vessel, basic water confidence and swimming proficiency, wearing of lifejackets by all staff, provision of water resistant cell phones, and use of safety rails or harnesses. Biomass should be gathered with buckets or nets that are tethered to the boat so that workers can avoid leaning over the side of their boat - a dangerous activity in this regard. At least two workers should be deployed for any bloom harvesting excursion, with one staffer at all times not directly exposed to scum material and thus available to summon and provide assistance should it be required.

### Cutaneous exposure risks

Most of the known cyanotoxins are highly water soluble, so the risk of intoxication via the dermal route for workers handling cyanobacteria in waterbodies or growing laboratory cultures would appear to be negligible. As discussed below, this may not be the case during preparation of purified cyanotoxins if solvents that could increase skin permeability are used, e.g. dimethyl sulfoxide. However, some cyanotoxins, particularly the dermal irritant toxins - aplysiatoxin, debromoaplysiatoxin and the lyngbyatoxins - produced by some marine cyanobacteria (mainly *Lyngbya majuscula*) [19] represent an occupational risk of acute skin irritant effects. In addition, the highly water soluble toxin cylindrospermopsin, produced by several freshwater and brackish water cyanobacterial genera, has been shown to produce cutaneous irritant and delayed-contact hypersensitivity reactions in experimental animals exposed to high concentrations [20], and cylindrospermopsin-producing cyanobacteria have produced delayed-contact hypersensitivity reactions in a human volunteer study [21]. The skin sensitising potential of cyanobacteria is poorly understood and under-researched, but there are some convincing case reports of allergic reactions provoked by cutaneous exposure to freshwater cyanobacteria, and numerous anecdotal reports of acute skin eruptions following such contact [22]. While the incidence and prevalence of cyanobacteria-related hypersensitivity reactions are unknown, as a precautionary measure the use of waterproof gloves is recommended for all staff handling bulk cyanobacterial biomass. Workers who are extracting, processing and working with purified cyanotoxins are advised to wear skin protection because of the (albeit poorly understood and unquantified) potential for sensitisation.

### Inhalational exposure risks

The potential for deleterious health effects from inhalation of cyanobacteria and cyanobacterial products in field locations - marine and inland waters - is not well understood. Some workers [23-26] have suggested that inhalational exposure to toxic cyanobacterial products may have contributed to the acute illnesses, some severe, in two groups of British soldiers and sea cadets in 1989 [27] and 1996 [24]. The recruits were participating in canoeing exercises involving full immersion in cyanobacteria-affected waterbodies. However, assuming the reported illnesses (fever, malaise, sore throat, unproductive cough, diarrhoea, vomiting, abdominal pain, headache, pneumonia) were caused by exposure to toxic cyanobacteria, the signs and symptoms may have developed as a result of oral exposure to cyanotoxins. Ingestion of water was reported in both these incidents, which involved canoe capsizing trials [24,27]. The possibility of reflex swallowing associated with sudden immersion leading to exposure to toxic cyanobacteria by the oral route should be considered when reviewing those events.

A prospective epidemiological study of recreational exposure to cyanobacteria found a statistically significant increase in reporting of respiratory symptoms amongst study subjects exposed to recreational waters with high cyanobacteria biomass estimates when compared to those exposed to waters with low cyanobacteria levels [28]. However, respiratory symptoms were mostly mild in extent, and the study was not designed to link the reported incidence of symptoms with specific exposure routes.

Two recent prospective studies of recreational exposure to cyanobacteria were designed to examine inhalational exposure: study subjects wore personal air samplers, and high-volume air samplers were employed at study sites in order to estimate airborne microcystin (MC) exposures. Low concentrations of MCs were detected by these methods. Low microcystin concentrations were also detected in post-exposure nasal swabs taken from subjects in one study, though no MCs were found in blood samples [29]. No differences in symptom reporting between study and control subjects were identified in either study [29,30].

Experimental work with rodent models has shown that some purified cyanotoxins can efficiently gain access to the circulation via the bronchial tract [31,32], but such studies involved direct application of solubilised cyanotoxins to the nasal or tracheal mucosa, i.e. these were not inhalational exposure experiments. A study by Benson et al [33] of inhalational exposure over seven days in mice to microcystin-LR (MC-LR) did not demonstrate any adverse pathological effects on the liver, kidney or gastrointestinal tract that would be expected after parenteral exposure. Inflammatory and necrotic damage to the nasal epithelium was the sole pathological finding [33]. The authors estimated daily exposures of 50 μg/kg body weight in their mice exposed to the highest experimental concentrations, which is the commonly accepted mouse acute LD_50 _by the intraperitoneal route [34]. A conference abstract [35] reported lethal exposures to MC-LR aerosol in mice by a single 10 minute inhalation dose. The LC_50 _was calculated at 18 μg/L of air, which, for a 25 g mouse with a minute ventilation of 40 mL would equate to an exposed dose of 290 μg/kg. These findings support general toxicological principles with respect to exposure routes for water soluble toxins and the efficiency with which each route provides access to the circulation: parenteral exposures>inhalational>oral>dermal.

While the inhalational route for exposure to cyanotoxins may be hazardous in theory, and the limited experimental evidence may support such concerns, the implications for exposure to toxigenic doses of cyanotoxins in field settings depend also on the degree to which intracellular cyanotoxins or those found freely soluble in the water are able to be effectively aerosolised. Cyanotoxins can be aerosolised; experimental studies on microcystins have shown that a bubble-bursting mechanism that models wind-generated wave action can transfer the toxin into air [36]. However, translating this experimental finding into implications for field exposure is problematic and requires more research. We are not aware of compelling evidence, anecdotal or otherwise, that cyanotoxins present in wet sprays generated by wave action or powered water craft have caused intoxication via inhalation in humans, wild animals or livestock in freshwater systems.

There are a small number of intriguing, largely anecdotal reports of outbreaks of respiratory illness associated with (presumed) inhalation of cyanobacterial products that have contaminated reticulated supplies and have aerosolised in hot water, as well as some preliminary epidemiological investigations into cyanobacteria in saunas [22,37]. The relationship to contaminating cyanobacteria in these incidents is tenuous, and considerably more research investigations are needed in order to ascertain the risks from cyanotoxins atomised in hot water. These reports presumably also bear little relevance when considering occupational exposures in open waterbodies.

Some toxic marine cyanobacteria may present a different picture with respect to inhalational exposure risks. We discuss some related issues below, though again the whole topic of aerosolised cyanotoxins is poorly understood.

Toxic *Lyngbya majuscula *in coastal waters may represent a rather specific hazard, as there are several anecdotal reports and outbreak-initiated investigations of non-bathers at beachside locations suffering respiratory and ocular symptoms that were temporally related to the presence of *L. majuscula *blooms [19]. An outbreak of upper respiratory and eye symptoms occurred amongst small business employees and visitors at a waterfront precinct in Hawaii in 1983. While not definitively confirmed, the causative agent was thought to be aerosolised products of a *L. majuscula *bloom that coincided with high tides and big surf generated by a tropical storm [38].

A cursory mention of occupational respiratory symptoms associated with harvesting *Trichodesmium *biomass by plankton net is made in a report discussing suspected toxic properties of this pelagic marine cyanobacterium. The report does not discuss prevailing weather and wave conditions when their *Trichodesmium *(predominantly *T. thiebautii*, with some *T. erythraeum*) was collected, so it is difficult to assess the degree to which filaments may have been aerosolised [39]. The toxicity of pelagic marine cyanobacteria (i.e. planktonic open-ocean species, as distinct from benthic coastal zone forms) is a poorly-understood and under-researched topic, though there are growing suspicions that several planktonic forms are capable of producing toxins. A paper from Brazil in 1963 discussed earlier reports of mass outbreaks of a febrile respiratory illness attributed to onshore blooms of *Trichodesmium *[40]. However, we are not convinced that this disease, named "Tamandaré Fever", might not have been equally explained by exposure to unidentified or mis-identified toxic blooms of the marine dinoflagellate *Karenia brevis*. Brevetoxins, the cyclic polyether toxins produced by *K. brevis*, are capable of being aerosolised to irritant and sensitising concentrations and can cause acute respiratory symptoms in beach workers and visitors [41,42]. For a recent review of the toxicity of marine cyanobacteria and discussion of Tamandaré Fever, see Stewart & Falconer [2].

Because of the lack of evidence for harmful effects via inhalation from harvesting cyanobacterial biomass from marine or fresh waters, we do not currently recommend the use of protective respiratory apparatus for such work. These recommendations should, however, be subject to revision if and when plausible reports of hazardous field conditions are published. We suggest that wherever possible such tasks should be scheduled at locations and in weather conditions that minimise spray generation, i.e. on calm waters undisturbed by powered watercraft. Harvesting should only take place when prevailing wind, wave and tidal conditions are such that spray generation is minimised. Such conditions are necessary requirements for harvesting surface scums of planktonic cyanobacteria that would otherwise be dispersed in choppy waters. Harvesting littoral zone benthic cyanobacteria such as *L. majuscula *from beaches or rock ledges in strong surf conditions is fundamentally unsafe in any case, due to the risk of imbalance and potentially catastrophic immersion.

Workers harvesting bulk cyanobacterial biomass need to be particularly cautious when dealing with senescent bloom material that may be subject to anaerobic decomposition, because of the potential for liberation of hazardous concentrations of hydrogen sulfide. A recent incident in France highlighted the potential for misadventure here; an extensive overgrowth of *Ulva lactuca *(sea lettuce, an otherwise non-toxic and indeed edible chlorophyte macroalga) reportedly resulted in the death of a horse and its rider being rendered unconscious after being overcome by H_2_S. Concern was also raised that the sudden death of a truck driver working to remove rotting biomass may also have been related to H_2_S intoxication, but this was apparently unconfirmed at the time [43].

### Harvesting and handling dry cyanobacterial biomass

The previous discussion and recommendations relate to the collection of wet cyanobacterial biomass, i.e. harvesting aquatic or marine toxic cyanobacteria directly from the environments in which they grow. The potential for inhaling harmful amounts of cyanobacterial products in field conditions increases where desiccated cyanobacterial biomass is harvested from shorelines and beaches, and occupational health and safety recommendations should be revised accordingly. Collecting such material may unavoidably generate dust which poses an inhalation hazard. Harvesting dry *Lyngbya majuscula *from beaches provides a case in point. Lyngbya-related toxins may be expected to cause acute respiratory symptoms if sufficient quantities of toxic dust are inhaled. Local authorities in south-east Queensland, where severe *L. majuscula *blooms occur regularly in coastal waters and embayments, mandate the use of protective respiratory apparatus for their staff that are responsible for the collection and disposal of cyanobacterial biomass that has washed up on beaches [44]. Dry toxic *L. majuscula *should be considered a particular hazard, though we suggest that prudent precautionary principles dictate that for all cyanobacteria, harvesting wet cyanobacterial biomass should, wherever possible, be favoured over collection of dry material. Light wetting of the required amount of desiccated material with a watering-can prior to collection may be all that is required to prevent dust being liberated; if collection of dry biomass is considered necessary, we recommend the use of respirator masks.

### Mass culture of toxic cyanobacteria in the laboratory

**Figure 2:**
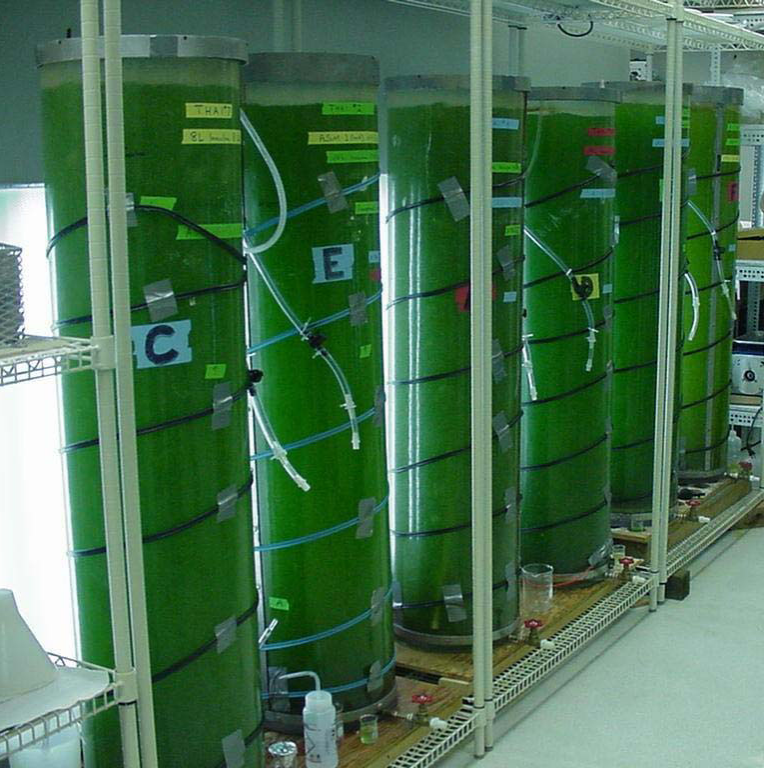
**Large-scale laboratory culture of *Cylindrospermopsis raciborskii***. Photo by author WWC.

Many scientists procure sufficient quantities of cyanotoxins for their research by growing cyanobacterial isolates in their laboratory. Cyanobacteria are grown using inorganic media and various techniques can be employed depending on the amount of toxin required and the available facilities. Simple methods may involve batch culture in a 5 or 10 litre flask using ambient light and temperature and intermittent aeration, which should allow for production of microgram to milligram quantities of cyanotoxins. More efficient systems may feature a dedicated growth chamber to allow temperature and illumination to be controlled; continuous culture vessels may be aerated and supplemented with additional CO_2_. Large-scale systems may have capacities of over 1,000 litres. When volumes of this size are being grown extra caution needs to be exercised in order to contain any spills and to prevent aerosols from being generated. Vented air from cultures should be passed over an acidified hypochlorite solution to inactivate any cells and cyanotoxins. Figure 2 shows a mass culture of toxigenic cyanobacteria in operation.

Occupational safety guidelines for laboratory production of toxigenic cyanobacteria should focus on avoiding contact with skin and mucous membranes. Gloves should be worn when handling culture vessels and equipment, and eye protection should be considered for procedures that may involve splash generation, e.g. when cleaning equipment or pouring out vessel products. As laboratory mass culture of cyanobacteria is conducted in closed systems, oral and inhalational exposures are not considered hazardous for such work, provided routine microbiological procedures are used when pipetting inoculum and vented air is contained and decontaminated. Aeration should be turned off five minutes before opening culture vessels so that aerosols can settle.

Contingency protocols should be established to deal with spills; disrupted integrity of a large-scale culture system could potentially result in the release of large volumes of toxic material, so design features to minimise the likelihood of spillage such as isolation valves and secondary containment may be pertinent. Every attempt should be made to recover spillage and, if possible, extract usable concentrations of cyanotoxin. In the recovery process, aerosols may be generated if high-capacity pumps are used to transfer bulk culture material from the floor to the open top of a temporary or repaired containment vessel. Following cleanup of liquid spills the affected area should be decontaminated with hypochlorite (5-10% v/v).

Culture vessels should be identified as containers for toxic liquids by tagging with biohazard labels. Information on the species being grown, toxin/s produced, and the location of specific protocols for managing accidental release should be clearly visible.

## Concentration, lyophilisation and shredding/powdering

Wild-harvested or laboratory cultured cyanobacteria will usually be concentrated and/or lyophilised for storage and subsequent solvent extraction. Concentration of planktonic cyanobacteria can be accomplished by either filtration or centrifugation. Both procedures will require skin protection (lab coat, nitrile or latex gloves) and safety glasses to prevent eye splash. Disposal of filtrates, supernatants and used culture media should be undertaken with consideration given to the prevention of environmental contamination by viable cyanobacterial cells (see below).

Lyophilisation of planktonic and benthic cyanobacteria may create an additional hazard potential for exposure by inhalation. Therefore subsequent processing of dry material will require precautions to minimise such exposures because of the potential risk of respiratory irritation (particularly from the *Lyngbya*-related toxins) and/or sensitisation. In the laboratory, this can be achieved by performing tasks involving lyophilised or naturally desiccated cyanobacteria in a fume cupboard. Small quantities may be shredded with a simple device such as a domestic coffee grinder; a swing mill is useful for larger amounts or to achieve a fine powder. Either way, transfer of ground material to storage containers should be done under a fume hood, ideally one that provides for low air movement over powdered material.

## Solvent extraction, sonication, filtration

Procedures involved in producing crude toxin extracts such as vacuum filtration, rotary evaporation, sonication and lyophilisation should be conducted with regard to preventing exposure to skin; the usual protective measures of gloves and lab coat should be worn. Eye splash protection should be considered for specific procedures, for example when positive pressure is applied to syringe filters and solid-phase extraction cartridges; significant force may be generated such that accidental disconnection of apparatus under pressure will increase the risk of an explosive spray. Disposal of cyanobacterial biomass remaining after solvent extraction should be undertaken with regard to preventing environmental contamination (see section below: "Environmental risk prevention"). Many water-soluble cyanotoxins will be extracted with water or methanol; some less polar toxins may be extracted with acetone or other solvent/s. Additional safety precautions relating to use of organic solvents should be considered when these are used for cyanotoxin extraction and purification. Material safety data sheets (MSDS) for specific solvents should be consulted for identification and prevention of additional hazards (e.g. flammability). Use of non-polar solvents may increase the possibility of dermal absorption of cyanotoxins; particular care should be taken to protect the skin from inadvertent exposure.

The final product of crude extraction preparation may, depending on the particular cyanotoxin, be a concentrated and potentially lethal mixture. While accidental oral exposure in a laboratory setting is a very remote possibility, vials and containers holding concentrated extracts should be clearly labelled and identified as toxic materials. Storage of concentrated cyanotoxin extracts, even though not yet purified, should from this stage be conducted with regard to preventing access by persons with criminal intent (see below for discussion of biosecurity provisions).

## Preparative and analytical HPLC; HPLC + mass spectrometry

Instrumental analytical procedures to further isolate, purify and quantify cyanotoxins will involve mixing cyanobacteria extracts with various organic solvents. Systems should be automated wherever possible to minimise contact as manual injectors may occasionally obstruct, in which case explosive sprays may occur. Use of an automated fraction collector should mitigate inhalation risks; if fractions are collected manually care should be taken to ensure that the eluate does not aerosolise. MSDS and occupational safety requirements for the specific chromatographic solvents should also be consulted.

## Lyophilisation, reconstitution and/or storage of final product

Quantified, pure cyanotoxin product will be lyophilised and stored as a solid or reconstituted in a suitable solvent for storage. Particular care should be taken when handling glass lyophilisation vessels; pure, dry cyanotoxins have a low density and are readily dispersible such that accidental breakage of a vessel in an enclosed space would present a risk of inhalation exposure. Contamination of laboratory floor and bench spaces by lyophilised cyanotoxins will present hazards to housekeeping staff as well as research and technical workers.

## Decontamination of glassware before routine cleaning

In many laboratories, routine collection and cleaning of reusable glassware is conducted by relatively unskilled staff. These workers should not be expected to be aware of the specific risks associated with exposure to highly toxic materials like cyanobacteria. It should therefore be the responsibility of laboratory staff working with cyanobacteria and/or cyanotoxins to decontaminate used glassware before sending it for cleaning. Residues remaining on glassware after preparation of crude extracts or purified products can in most instances be safely decontaminated by rinsing with a solvent such as acetone or ethanol, followed by a water rinse.

## Use of purified cyanotoxins for research purposes

Purified cyanotoxins may be used for a wide variety of research purposes, e.g. determination of physico-chemical and structural properties, as functional analogues for various metabolic processes, e.g. anatoxin-a as a nicotinic agonist [45]; microcystin-LR as a protein phosphatase inhibitor [46,47], in basic toxicology, toxicokinetics and toxicodynamics experiments, ecotoxicology studies and for investigation of pharmacological properties. Some cyanotoxins are available as standard reference materials for analytical chemistry work, so laboratories conducting research and/or routine monitoring work for cyanotoxins in waters and biota will also use pure cyanotoxins. Some cyanotoxins (several microcystin congeners, cylindrospermopsin, saxitoxin, neo-saxitoxin, nodularin, anatoxin-a) are available commercially in microgram quantities. MSDS are available for these toxins from some suppliers' websites. Certified reference standards (and MSDS) for cylindrospermopsin and several saxitoxin analogues are supplied by the Canadian National Research Council's Institute for Marine Biosciences, see [48]. Researchers and scientists working with purified cyanotoxins on various physico-chemical or *in vitro *projects should consult the appropriate MSDS for occupational safety information on the specific cyanotoxin, cyanotoxin group and/or cyanotoxin/solvent mixture that most closely matches the particular cyanotoxin being studied. Researchers working with uncharacterised cyanotoxins or purified novel cyanobacterial compounds should consider the general advice in this paper and published MSDS, particularly regarding protection of skin and eyes and avoiding inhalation of dry product dusts. The remaining discussion on occupational health and safety matters pertains to cyanotoxin work on animal models.

### Use of pure cyanotoxins for *in vivo *research

Toxicological and pharmacological research on cyanotoxins based on animal models may present some particular risks both for the researcher and staff working in the animal research institution. Specific risks will depend on the exposure route being investigated, the size of the animals and the concentrations of cyanotoxin being administered. Most cyanotoxin research to date has been conducted on small rodents dosed by various parenteral routes. This reflects one of the main difficulties with *in vivo *cyanotoxin research: cyanotoxins in general are difficult or expensive to procure, so there has been a bias towards the most efficient use of a scarce resource [49]. Dosing of small animals by the most direct exposure route generates the most information on toxicological effects from a toxin dose per unit of body weight basis. There is therefore a relative dearth of experimental research investigation into the toxicology of cyanotoxins in non-rodent and larger animal models and in all models by oral, inhalational and cutaneous/dermal exposure. Such investigations should and hopefully will be undertaken in future; appropriate occupational safety protocols will need to accompany these endeavours.

Dosing experimental animals with cyanotoxins by parenteral exposure routes opens the possibility of accidental injection into research workers. While such an event should be considered unlikely, the hazard does exist and is most significant in the case of experimental studies using large animals, when researchers may be handling doses that are highly toxic or even lethal for humans. Test animals will then need to be suitably restrained when syringes and hypodermic needles are used to administer cyanotoxins.

Inhalational exposure studies will normally be conducted in an enclosed chamber. Experimental design protocols should detail measures to eliminate risks to research and animal facility staff from accidental and incidental exposure to aerosolised cyanotoxins.

Cutaneous or dermal exposure studies are likely to use carrier vehicles other than water, i.e. relatively less polar solvents such as ethanol, methanol or acetone that will allow the chemical of interest to partition across cutaneous and epidermal tissues. Use of less polar vehicles will thus place researchers at greater risk of dermal exposure to water-soluble cyanotoxins that would otherwise not be expected to penetrate the skin to any significant extent. Cyanotoxins dissolved in the polar aprotic solvent dimethyl sulfoxide (DMSO) will present a particular dermal exposure hazard. Lethal intoxications by the dermal exposure route have been reported for both microcystin-LR and saxitoxin in DMSO administered to guinea pigs [50].

Experimental oral dosing of cyanotoxins will usually be accomplished by gavaging aqueous solutions or by adding known quantities of cyanotoxin to drinking water supplies. Feeding experiments to investigate trophic transfer of cyanotoxins or dietary transfer efficiency of contaminating cyanotoxins (e.g. from cyanobacteria-based nutritional supplements containing contaminant toxigenic cyanobacteria, or market garden produce spray irrigated with cyanotoxin-contaminated water) are conceivable. Oral exposure studies should, at the design stage, consider the potential for cyanotoxins in food or water supplies to be dispersed by experimental animals, and the attendant occupational safety risks for research and animal facility staff. Dry feed (e.g. containing cyanotoxin-contaminated fish meal) may be subject to dust formation by active animals. From an occupational health perspective, cyanotoxin feeding studies should, if possible, be conducted using moist feed. Contingency protocols for cleaning and disposal of spilled food and water should be applied.

Main excretion routes for the various cyanotoxins are via urine and faeces; proportional elimination by each route varies according to the specific cyanotoxin. The urinary system is the principal excretion pathway for cylindrospermopsin [51], whereas the microcystins (at least those variants that have been studied in this context) are mainly eliminated through the bile and then into faeces [13]. Saxitoxins are eliminated in urine and faeces [52,53]. Researchers and animal house staff collecting or disposing of urine from cyanotoxin-dosed animals should apply procedures to minimise aerosol formation. Latex or nitrile gloves should be worn at all times when handling excreta; faeces and carcases should be disposed of by incineration.

The general hazards outlined in the above discussion, and recommended management practices, are summarised in Table 1.

**Table 1:** Summary of specific hazards encountered when processing toxic cyanobacteria to produce cyanotoxins, and recommended control measures

Procedure	Hazard	Control measure
Bulk harvesting - wet biomass	Oral exposure	Avoid accidental immersion
	Cutaneous and mucous membrane exposure	PPE: waterproof boots, gloves, waders; avoid splash generation
	Inhalation exposure	Work in calm water condition
Bulk harvesting - dry biomass	Cutaneous and mucous membrane exposure	PPE: gloves, overalls
	Inhalation exposure	Consider wetting prior to harvest, otherwise protective respirator mask
Laboratory culture of toxic cyanobacteria	Cutaneous and mucous membrane exposure	PPE: gloves, lab coat, eye splash protection for specific procedures. Contingency procedures to manage accidental spillage
	Inhalation exposure	Discontinue aeration for 5 minutes before opening culture vessels
Concentration, lyophilisation, powdering	Cutaneous and mucous membrane exposure	PPE: gloves, lab coat, eye splash protection
	Inhalational exposure	Low-flow fume cupboard
		PPE: face mask
Solvent extraction, sonication, filtration, chromatography,	Cutaneous and mucous membrane exposure	PPE: gloves, lab coat, eye splash protection for specific procedures
	Inhalational exposure	PPE: face mask for procedures affording risk of spray generation
Lyophilisation of pure product	Cutaneous exposure	PPE: gloves, lab coat
	Inhalational exposure	Care to avoid vessel breakage
		PPE: face mask
Toxicity studies using *in vivo *models	Parenteral exposure	Care when handling hypodermic needles, restraint of animals for dosing
	Cutaneous exposure	PPE: gloves, lab coat
	Inhalational exposure	Procedures to minimise or eliminate aerosol formation when disposing of urine. Experimental design features to avoid dry dust dispersal in feeding studies
		PPE: face mask if aerosols likely

## Other occupational safety matters relating to cyanobacteria and cyanotoxins

• **Radiolabelled toxin: **Some researchers may grow toxigenic cyanobacteria in media with an added ^14^C source in order to produce labelled cyanotoxin for toxicokinetics studies or other research endeavours. Workers producing and using radiolabelled cyanotoxins will need to prepare occupational safety protocols that consider safe use and disposal of such toxins. Such protocols should reflect the risks associated with exposure to both the cyanotoxin and the radioisotope. Animal house staff will need to be aware of the potential for labelled toxin excreted in the urine of experimental animals to be aerosolised.

• **Use of toxic cyanobacteria (i.e. not purified toxins): **Workers from varying research backgrounds (e.g. genetics, ecology) may work directly with toxigenic cyanobacteria rather than purified cyanotoxins. Toxicology researchers may also work directly with crude or partially purified extracts of toxic cyanobacteria in order to overcome difficulties relating to availability of purified toxins. This has advantages related to quantity of supply, especially if the amounts of toxin required are high. An example of this is an oral toxicity trial carried out in pigs with *Microcystis *bloom material, which involved collection and processing of several thousand litres of cyanobacterial scum [54]. Occupational health risks from this exercise included skin contact and inhalation risk in scum collection, mixing, sub-sampling and supply to the animals. In the near future it is likely that a major cyanobacterial processing effort will be required in order to supply the quantities of toxin needed for extended oral toxicity trials in rats or dogs, which will form an essential part of carcinogenicity risk assessment. And a recent recommendation has been made for clonal cultures of cyanobacteria that have been characterised with respect to toxin production to be made available to toxicology researchers internationally to allow for inter-laboratory comparison of findings [49]. Contact with cyanobacterial blooms or extracts may involve risks from components other than the toxin under investigation, due to the wide range of bioactive materials in cyanobacteria [55].

The discussion in this paper on the various health risks and safety strategies for scientific and technical staff working on purified cyanotoxins may be applied to tasks involving handling of toxigenic cyanobacteria and various extracts from same. However, further considerations relating to disposal of potentially viable cyanobacterial cells should apply, and are discussed in the following section.

## Environmental risk prevention

Invasive microalgal incursions causing significant economic and ecological impacts have been reported from both marine and freshwater environments. Ballast water escapes of toxic marine dinoflagellates causing outbreaks of human saxitoxin poisoning in new geographic regions have been reported [56]. Invasions of the freshwater diatom *Didymosphenia geminata *in New Zealand rivers have caused widespread ecological damage, adverse affects on recreational fisheries and fouling of water off-take structures [57]. It is important that researchers working with living or viable cyanobacteria do not contribute to the problem of feral microalgae when disposing of spent culture material or harvested bloom material. Inter- and intra-national movement of live cyanobacteria occurs across several and varied research endeavours, e.g. toxicology, ecology, genetics, pharmacology and natural products discovery. Indeed, for cyanotoxin researchers a recent recommendation has been made to expand inter-laboratory availability of clonal cyanobacterial cultures [49].

Customs and quarantine clearances covering importation of live cyanobacteria should incorporate protocols for safe disposal of solid and liquid waste containing live or potentially viable cells. However, transport and use of live cyanobacteria within national borders will not be subject to any regulatory oversight, so the onus will be on individual researchers and institutions to take responsibility for the safe environmental disposal of native cyanobacteria. Translocation of "native" biota from outside its natural range can result in catastrophic ecological consequences, in much the same way as the establishment of intercontinental feral pests. Australia, for example, has some particularly troubling experiences with the inter-regional invasion of native plants [58]. The invasive potential of cyanobacteria is essentially unknown, but a precautionary approach by research workers in this regard is imperative. Cyanobacteria can produce a wide array of biologically active metabolites, some of which are toxic to humans and animals. Allelopathic properties may be a particular ecological hazard, should accidental escapes become successful bioinvasions [59].

Solid waste cyanobacteria should be incinerated. Liquid wastes may be autoclaved at 121°C for 15-20 minutes in the case of small volumes; up to 60 minutes for volumes up to 10 L. If a suitably sized autoclave is not available or large volumes (>10 L) of spent growth medium are to be disposed of, a chemical oxidant should be used to ensure that all cyanobacterial cells are rendered non-viable. Chlorine bleach is widely used for large liquid volumes which require sterilisation as it is effective in rendering cells non-viable but may not kill all specialised cells such as cysts (in the case of eukaryotic microalgae) [60]. Applying this understanding to cyanobacteria, it may be appropriate to assume that bleach may not destroy all akinetes, though it seems that the specific topic of cyanobacterial akinete eradication has not been well researched. Normal application rates are 1-5 mL of commercial bleach per litre of liquid, with several hours reaction time required (this can vary depending on the concentration of organic matter and exposure to sunlight during treatment). Shorter reaction times can be achieved with higher concentrations of bleach. Neutralisation of the treated solution with a reducing agent (1 mL of 25%w/v sodium thiosulfate solution per 4 mL of bleach) is required before disposal [60].

Acidified hypochlorite has a stronger antimicrobial action than stable bleach due to an increased concentration of dissociated HClO. Addition of acetic acid to hypochlorite solutions to pH 5.0 provides a highly effective bactericide [61], though additional occupational safety considerations will need to be observed regarding the potential for liberation of chlorine gas from hypochlorous acid. Acidified hypochlorite can be added to liquid waste prior to disposal to make a 5-10% v/v concentration; contact time with hypochlorite should be at least 30 minutes, and chlorinated wastes should be further diluted before disposing into domestic wastewater systems. Copious volumes of liquid waste that might conceivably be generated by (for example) a large animal feeding study may need to be handled by a professional liquid waste disposal agency.

Culture vessels and any accessory laboratory equipment that has had contact with viable cyanobacteria should be decontaminated (soaked, ideally) with hypochlorite solution before being rinsed and cleaned. Autoclavable laboratory equipment should be heat sterilised. Field sampling equipment should likewise be decontaminated between use at different sites and prior to storage.

## Biosecurity and bioterrorism precautions

As many cyanobacterial products are highly potent toxins, a number of these compounds have come to the attention of national security agencies in some countries because of their potential to be used as chemical weapons. Saxitoxin is scheduled as a toxic chemical under the Chemical Weapons Convention; saxitoxin and microcystin are on the Australia Group list of biological agents for export control [62,63]. Research facilities that make, acquire and use cyanotoxins may be subject to regulatory oversight regarding production, storage and transport of these materials [64]. Researchers who are not currently the focus of such scrutiny might anticipate some changes in this regard, so it may be prudent to establish and maintain pre-emptive protocols to document the quantities of various cyanotoxins that are purchased, produced and used, and to establish secure storage and laboratory facilities. Restricted access may be a requirement of police and/or security agencies when pure cyanotoxins are produced in multimilligram quantities. Safeguards are necessary to prevent theft and diversion for nefarious purposes, though some workers have noted that recent regulatory concerns have impeded legitimate research activities, and have called for provisions for safe transport and use in secure laboratories [48].

## Conclusion

Exposure to cyanotoxins via oral, inhalational or cutaneous exposure routes during harvesting or production, extraction and purification will depend on the specific task being conducted. Harmful consequences of such exposure can be expected to vary according to the specific cyanotoxin encountered, as well as the usual dose-related considerations. Common to all occupational health and safety strategies, the efficacy of protection measures will depend on both the appropriate choice of intervention and its acceptance by laboratory staff. These aims may be achieved through education programs to identify potential hazards from particular task-related exposure pathways, discussing risks and attendant amelioration measures, and documenting revisions to such programs. Identification of hazards to ancillary laboratory workers and measures to prevent the introduction of viable cyanobacterial cells to the external aquatic environment should be essential considerations.

Personal protection should be maintained at all times to minimise human exposure risk during collection of bloom material, mass culture production, toxin extraction, purification and quantitation, *in vivo *applications and live culture disposal. General laboratory personal protection encompasses the wearing of laboratory coats, appropriate gloves (eg: nitrile gloves for solvent exposure), safety glasses (splash protection), face masks (when potentially exposed to sprays, powders and dried toxic material) and handling of toxic material in fume hoods. Access to chemical alert databases and material safety data sheets is mandatory and the creation of standard operating procedures for general occupational safety including procedures for spillages is strongly recommended.

## Abbreviations

DMSO: dimethyl sulfoxide; HPLC: high performance liquid chromatography; LC: lethal concentration; LD: lethal dose; MC: microcystin/s; MSDS: material safety data sheet/s; PPE: personal protective equipment

## Competing interests

The authors declare that they have no competing interests.

## Authors' contributions

IS prepared the initial draft. IS, WWC, RS, GKE, WAW, AAS and GRS wrote the occupational safety guidelines. IS, GBM and KR prepared the environmental hazard guidelines. All authors read and approved the final draft.
